# Comprehensive QTL analyses of nitrogen use efficiency in *indica* rice

**DOI:** 10.3389/fpls.2022.992225

**Published:** 2022-09-23

**Authors:** Xiuyan Liu, Hong Jiang, Jing Yang, Jiajia Han, Mengxian Jin, Hongsheng Zhang, Liang Chen, Sunlu Chen, Sheng Teng

**Affiliations:** ^1^College of Material and Environmental Engineering, Hangzhou Dianzi University, Hangzhou, China; ^2^Laboratory of Photosynthesis and Environmental Biology, CAS Center for Excellence in Molecular Plant Sciences, Shanghai Institute of Plant Physiology and Ecology, Chinese Academy of Sciences, Shanghai, China; ^3^State Key Laboratory of Crop Genetics and Germplasm Enhancement, Jiangsu Collaborative Innovation Center for Modern Crop Production, Jiangsu Province Engineering Research Center of Seed Industry Science and Technology, Nanjing Agricultural University, Nanjing, China; ^4^Shanghai Agrobiological Gene Center, Shanghai, China

**Keywords:** nitrogen-use efficiency, quantitative trait locus, effective panicle number, QTL-by-environment interaction, QTL cluster, *Oryza sativa*

## Abstract

Nitrogen-use efficiency (NUE) in rice is a complex quantitative trait involved in multiple biological processes and agronomic traits; however, the genetic basis and regulatory network of NUE remain largely unknown. We constructed a high-resolution microarray-based genetic map for 261 recombinant inbred lines derived from two *indica* parents. Using 2,345 bin markers, comprehensive analyses of quantitative trait loci (QTLs) of seven key agronomic traits under two different N levels were performed. A total of 11 non-redundant QTLs for effective panicle number (EPN), 7 for grain number per panicle, 13 for thousand-grain weight, 2 for seed-setting percentage, 15 for plant height, 12 for panicle length, and 6 for grain yield per plant were identified. The QTL regions were as small as 512 kb on average, and more than half spanned an interval smaller than 100 kb. Using this advantage, we identified possible candidate genes of two major EPN-related QTLs. One QTL detected under both N levels possibly encodes a DELLA protein SLR1, which is known to regulate NUE, although the natural variations of this protein have not been reported. The other QTL detected only under a high N level could encode the transcription factor OsbZIP59. We also predicted the possible candidate genes for another three of the NUE-related QTLs. Our results provide a reference for improving NUE-related QTL cloning and promote our understanding of NUE regulation in *indica* rice.

## Introduction

As one of the major staple cereals, the yield of rice (*Oryza sativa*) largely depends on the substantial supply of nitrogen (N) fertilizers. The large application of N fertilizers has not only increased the economic cost of farming, but has also caused severe environmental degradation (Guo et al., 2010; Chen et al., 2014; Lu et al., 2015). Improving the nitrogen-use efficiency (NUE) of rice as well as other crops is thus an important research topic, especially considering the increased requirements for green and sustainable agriculture (Tilman et al., 2002; Luo et al., 2020; Sandhu et al., 2021). The breeding of high-NUE varieties is key to reducing the input of N fertilizers, and this strategy requires the identification and application of elite alleles responsible for high NUE (Ali et al., 2018; Hawkesford and Griffiths, 2019; Liu et al., 2022).

NUE is a complex characteristic and quantitative trait involved in N sense, uptake, transport, reduction, assimilation, and signaling as well as the crosstalk between N and other nutrients (Fan et al., 2017; Lee, 2021). Differences in NUE (and N levels) impact various morpho-agronomic and physiological traits, including tiller number (TN), effective panicle number (EPN), spikelet number, 1,000-grain weight (TGW), plant height (PH), grain yield per plant (GYPP), leaf color, and dry weight of the shoots and roots (Ali et al., 2018; Sandhu et al., 2021). Analyses of quantitative trait loci (QTLs) controlling NUE in rice have been performed using various traits or characteristics as indicators based on different biparental populations as well as natural populations (Tong et al., 2006; Cho et al., 2007; Senthilvel et al., 2008; Wang et al., 2009; Wei et al., 2012; Yue et al., 2015; Nguyen et al., 2016; Zhou et al., 2017; Bai et al., 2021; Lv et al., 2021; Rakotoson et al., 2021; Shen et al., 2021; Xin et al., 2021; Li et al., 2022).

Recently, several QTLs involved in NUE were cloned and the related molecular mechanisms were explored in rice. The gene *qNGR9,* which is related to the response of PH and TN to N levels, encodes a γ subunit of G proteins, which is an allele of *DEP1* previously known to regulate panicle architecture (Huang et al., 2009; Sun et al., 2014). DEP1 interacts with the α (RGA1) and β (RGB1) subunits of G proteins, and a lack of DEP1 inhibits N responses in rice (Sun et al., 2014). The NH_4_^+^ uptake rate-related QTL *qNGR2* encodes the transcription factor GRF4, which regulates various genes involved in ammonium transport and assimilation as well as carbon fixation (Li et al., 2018). The NO_3_^−^ uptake-related QTL *qDNR1* encodes a methionine-specific aminotransferase regulating auxin homeostasis and thus induces auxin response factor-mediated activation of nitrate uptake and metabolism genes (Zhang et al., 2020). The chlorate (toxic analog of nitrate) sensitivity-related QTL *qCHR10* encodes a nitrate-transporter NRT1.1B, which contributes to the differences in nitrate-absorption activity between *indica* and *japonica* rice, and the *indica* allele increases the activity of nitrate uptake and root-to-shoot transport (Hu et al., 2015). An NADH/NADPH-dependent NO_3_^−^ reductase NR2 encoded by the chlorate resistance-related QTL *qCR* enhances nitrite uptake *via* a feed-forward interaction with NRT1.1B (Gao et al., 2019b). A genome-wide association study (GWAS) of PH, EPN, and YPP under different N levels identified a nitrate transporter OsNPF6.1 and an NAC transcriptional factor OsNAC42 involved in NUE, and the latter directly binds to the promoter of the former to active its expression (Tang et al., 2019). Another GWAS of the TN response to N levels identified a transcription factor OsTCP19 that regulates the expression of the tiller-promoting gene *DLT* (Liu et al., 2021). A 29-bp deletion in the promoter of *OsTCP19* was responsible for high NUE by impacting the repression effect of OsLBD37 and OsLBD39 proteins on the promoter (Liu et al., 2021).

Although numerous NUE-associated QTLs and multiple causal genes have been identified, the NUE regulatory network remains largely unknown considering the complex involvement of NUE in different biological processes and agronomic traits. Here, we genotyped a population of recombinant inbred lines (RILs) using single-nucleotide polymorphism (SNP) microarrays to generate a high-resolution genetic map, which we used for comprehensive QTL analyses of seven key agronomic traits [EPN, grain number per panicle (GNPP), TGW, seed-setting percentage (SSP), PH, panicle length (PL), and GYPP] related to NUE under low N (LN) and high N (HN) levels. We identified 33 and 26 QTLs under LN and HN conditions, respectively, as well as 57 multi-environment QTLs and eight ratio trait-related QTLs. We also observed six QTL clusters located in five chromosomes and examined the possible candidate genes of two EPN-related QTLs (located in two different QTL clusters)—one detected under both LN and HN conditions and the other under only HN. Our results provide new genetic insights into the NUE regulatory network in *indica* rice, and the identified narrow regions of QTLs could promote the cloning of causal genes in the future.

## Materials and methods

### Plant materials and field experiment

An RIL population (F11:12 generation) consisting of 261 lines derived from *O. sativa ssp. indica* cv. Zhanshan97 (ZS97) and HZ5 was used in the study. ZS97 showed LN tolerance, whereas HR5 exhibited N sensitivity (Tong et al., 2011). The sterilized seeds were sowed in paddy fields at Songjiang, Shanghai, China; one-month-old seedlings were transplanted. Two replicates were performed for each condition. In each replicate, for each line, six rows of plants with a distance of 15 cm (six plants for each row, 10–12 cm interval) were planted. For LN conditions, 120 kg urea/ha was supplied, whereas for HN conditions, N fertilizer was supplied twice at 187.5 and 112.5 kg urea/ha. Excluding the different N levels, the plants under the two conditions were managed the same.

### Trait evaluation and analysis

The EPNs of 20 plants and GNPPs of three major panicles of 20 plants were counted for each line under each condition. The SSP was calculated as the percentage of the filled grains of the total GNPP. The TGW of each line was determined using 1,000 seeds in each of three repeats. The PHs of 20 plants and PLs of five panicles of 20 plants were measured. The GYPP was estimated using the following formula: (EPN × GNPP × TGW × SSP)/1,000. The ratio traits were relative ratios of the agronomic trait value under LN conditions to its counterpart under HN conditions for each of the agronomic traits. Correlation tests showed a significant correlation between each pair of replicates, and the final averages of two replicates of LN/HN conditions were used for analysis in this study. Correlation analysis and path analysis were performed using SPSS version 18.0 (IBM, NY, United States).

### Genotyping and linkage map construction

The genomic DNA of the young leaf samples of the RILs and their parental lines was extracted using the DNeasy Plant Mini Kit (QIAGEN, Germany). The Quant-iT dsDNA HS assay kit and Qubit fluorometer (Invitrogen, CA, USA) were used for quantitating double-stranded DNA in solution. Genotyping was performed on the Affymetrix GeneChip 3,000 platform using an updated version of the GeneChip Rice 44 K SNP Genotyping Array (Affymetrix, CA, United States) following the manufacturer’s protocol. Briefly, all DNA samples were normalized to 50 ng/μl. Then, 5 μl (250 ng) of double-stranded DNA was digested and ligated to adapters using T4 DNA ligase. Samples were then PCR-amplified using TITANIUM Taq polymerase (Takara, Japan) on an ABI9700 machine (Applied Biosystems, CA, United States), and PCR products were purified followed by fragmentation. Samples were then injected into cartridges, hybridized, washed, and stained. Mapping array images were obtained using the GeneChip Scanner 3000. Genotypes were called using BRLMM software (Affymetrix), and any sample with a genotype call rate < 95% was considered a quality control failure. After filtering, 47,892 qualified SNPs out of 50,281 SNPs were obtained. In total, 12,152 effective SNPs (polymorphic between parents and in the population) were used for map construction. To remove the redundant markers due to the high linkage disequilibrium, a sliding-window approach was used and consecutive 50-kb intervals that lacked a recombination event in the population were combined into bins. A total of 2,345 bin markers indicating recombination events across the whole population were identified and used for linkage map construction by JoinMap software version 2.0 (Stam, 1993).

### Comprehensive QTL analyses

The final averages of two replicates were used as the phenotypes for QTL analyses. Based on the linkage map constructed above, the QTL analyses were performed for LN and HN conditions as well as for ratio traits using the inclusive composite interval mapping (ICIM) model *via* the ICIM-ADD function in QTL IciMapping software version 4.2 (Meng et al., 2015). The multi-environment QTLs for QTL-by-environment interaction analysis were identified using the MET module in QTL IciMapping software. Default parameters were used except for a step of 0.1 cM. The threshold of logarithm of odds (LOD) for agronomic traits was set as 3.0, and that for ratio traits as 2.5. Major QTLs were defined in this study with a phenotypic variance explained (PVE) ≥ 10%. Positive and negative values of additive effect, respectively, indicate positive alleles from ZS97 and HR5.

### Candidate gene analysis

Before conducting candidate gene analysis for the examined QTLs, the RIL lines were divided into two groups according to the alleles of the QTL. The differences in the six traits between the two groups were compared for QTL confirmation. The IDs and annotations of the genes in the QTL regions were extracted from the MSU Rice Genome Annotation Project database and RAP-DB database (Sakai et al., 2013). The expression profile data were derived from the CARMO platform (Wang et al., 2015) and were visioned using a heatmap. Homology analysis between rice and *Arabidopsis thaliana* genes was carried out using BLASTP with protein sequences of rice genes against *Arabidopsis* protein sequences in the TAIR database (Berardini et al., 2015). For sequencing the candidate gene alleles of the parents, fragments were amplified using high-fidelity DNA polymerase KOD Plus Neo (Toyobo, Japan).

### Co-expression network analysis

The co-expression network analysis was performed using the RiceFREND database version 2.0 based on the gene expression data from RiceXPro (Sato et al., 2012a,b). The weighted Pearson’s correlation coefficients were calculated to reduce unsuitable effects, and the mutual rank was used as an index for co-expression. The top-50 co-expressed genes according to the mutual ranks were used for further analysis. Gene Ontology (GO) enrichment analysis was performed by gProfiler server (Raudvere et al., 2019). The GO items were considered enriched at a false discovery rate (FDR) < 0.05.

## Results

### Response of the RIL population and parents to different N levels

**Figure 1:**
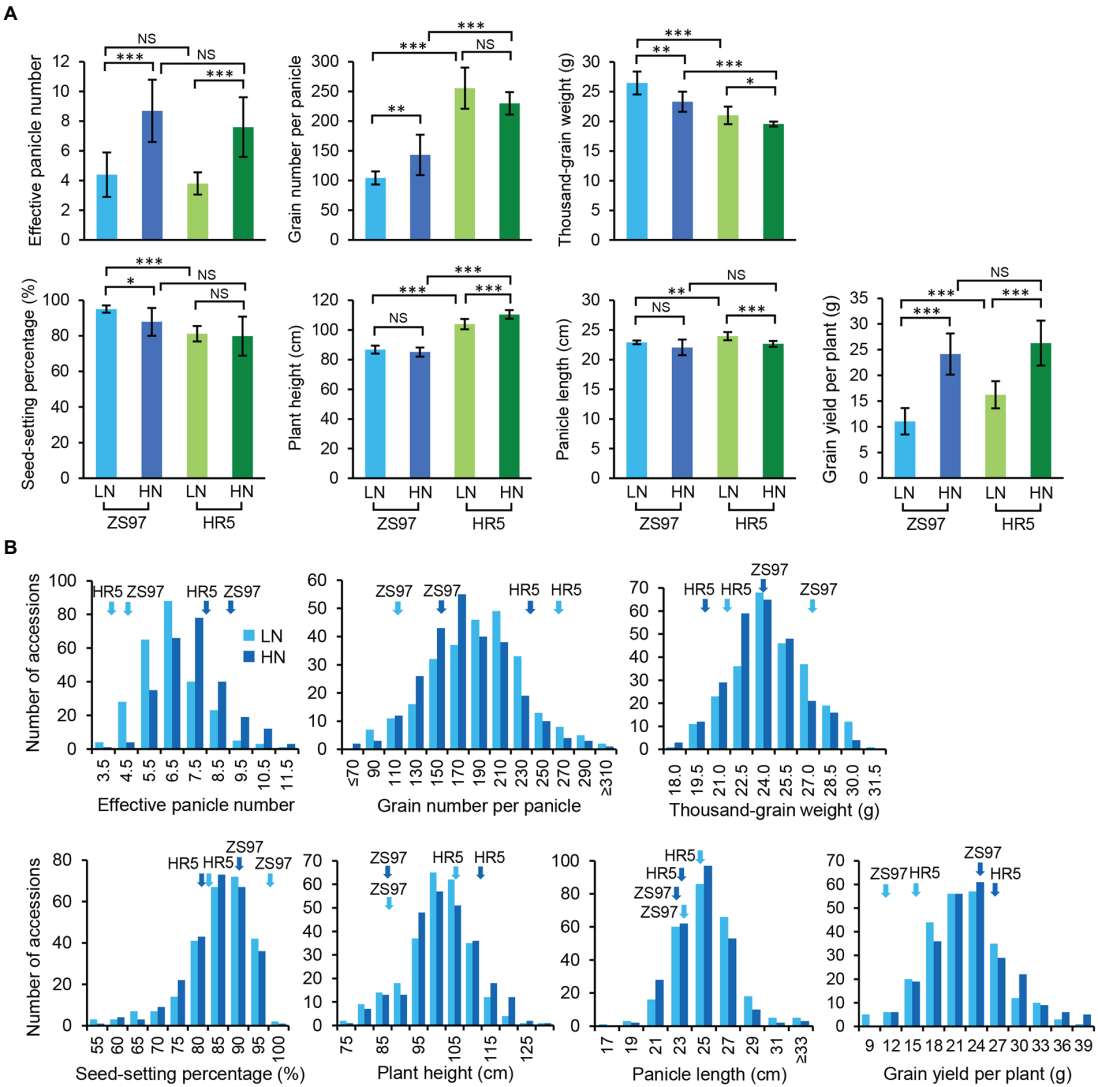
Performance of the agronomic traits of the parents **(A)** and RIL population **(B)** under two N levels. LN, low nitrogen; HN, high nitrogen. ^***^p < 0.001, ^**^p < 0.01, ^*^p < 0.05; NS, not significant. The light and dark blue, respectively, indicate LN and HN conditions. The performances of the parents are marked by arrows.

Under LN conditions, the parent ZS97 differed significantly from the parent HR5 for all examined agronomic traits (all *p* < 0.0012) except for EPN (*p* = 0.30). Under HN conditions, ZS97 only showed significant differences in GNPP, TGW, and PH compared with HR5 (all *p* < 7.25E-5; Figure 1A). The EPNs and GYPPs of both the parents increased in response to HN (*p =* 1.30E-4 and 6.01E-07 for ZS97; 2.47E-4 and 2.93E-05 for HR5); however, the TGWs of both decreased in response to HN (*p =* 0.0018 for ZS97, 0.018 for HR5; Figure 1A). Additionally, the different N levels also significantly impacted the GNPP and SSP of ZS97 (*p =* 0.0075 and 0.023) as well as the PH and PL of HR5 (*p =* 4.03E-4 and 3.08E-4; Figure 1A). These data suggest that the two parents showed distinct responses to different N levels, as reflected in the agronomic traits measured.

Under both LN and HN conditions, six of the agronomic traits of the RIL population displayed normal distributions, while SSP showed a skewed distribution (Figure 1B; Table 1). Different degrees of transgressive inheritance were observed for all the agronomic traits, especially for PL (Figure 1B). Strikingly, although both the parents exhibited a similar performance of EPN under either of the conditions, the RIL population showed extreme phenotypical segregation of EPN, which suggests that complementary but distinct genetic mechanisms control the EPN of the parents. One-way analysis of variance (ANOVA) revealed that the EPNs of the population between the two conditions showed the most significant differences (*p* = 9.56E-16), followed by TGW, GNPP, PL, and GYPP (all *p <* 0.020; *p* = 0.082 for PH, *p* = 0.50 for SSP). The results of the paired *t*-tests support significant differences between the two conditions for all the traits (all *p <* 0.004) except for SSP (*p* = 0.36). The difference in EPN values of the population between the two conditions also received the smallest *p*-value (5.70E-29), which is consistent with previous observations that an increase in tiller numbers is a major response to N levels (Li et al., 2018; Wu et al., 2020).

**Table 1:** Summary of seven agronomic traits of the RIL population under different N levels.

Trait^a^	Condition^b^	Mean	SD^c^	CV^d^	Minimum	Maximum	Ratio^e^	Skewness	Kurtosis
EPN	LN	5.99	1.33	0.22	3.30	10.90	3.30	0.68	0.89
EPN	HN	6.99	1.39	0.20	3.38	11.13	3.30	0.46	0.09
GNPP	LN	179.29	44.51	0.25	78.89	330.22	4.19	0.11	0.12
GNPP	HN	167.72	41.54	0.25	36.42	314.37	8.63	0.19	0.49
TGW	LN	23.89	2.58	0.11	17.78	30.11	1.69	0.13	−0.37
TGW	HN	23.17	2.45	0.11	16.16	29.80	1.84	0.18	−0.01
SSP	LN	82.72	8.21	0.10	51.28	95.93	1.87	−1.27	1.91
SSP	HN	82.29	7.75	0.09	51.36	96.00	1.87	−1.06	1.47
PH	LN	98.14	9.24	0.09	70.00	136.38	1.95	−0.16	1.12
PH	HN	99.42	9.65	0.10	71.88	126.00	1.75	−0.09	0.14
PL	LN	24.36	2.55	0.10	16.67	33.63	2.02	0.44	1.38
PL	HN	23.81	2.29	0.10	18.93	32.93	1.74	0.60	1.54
GYPP	LN	21.85	5.74	0.26	10.18	43.84	4.31	0.81	1.26
GYPP	HN	20.80	5.36	0.26	6.99	41.30	5.91	0.24	0.62

a EPN, effective panicle number; GNPP, grain number per panicle; TGW, thousand-grain weight; SSP, seed-setting percentage; PH, plant height; PL, panicle length; GYPP, grain yield per plant.

b LN, low nitrogen; HN, high nitrogen.

c SD, standard deviation.

d CV, coefficient of variation.

e Ratio, maximum/minimum ratio.

### Correlation among different agronomic traits

**Figure 2:**
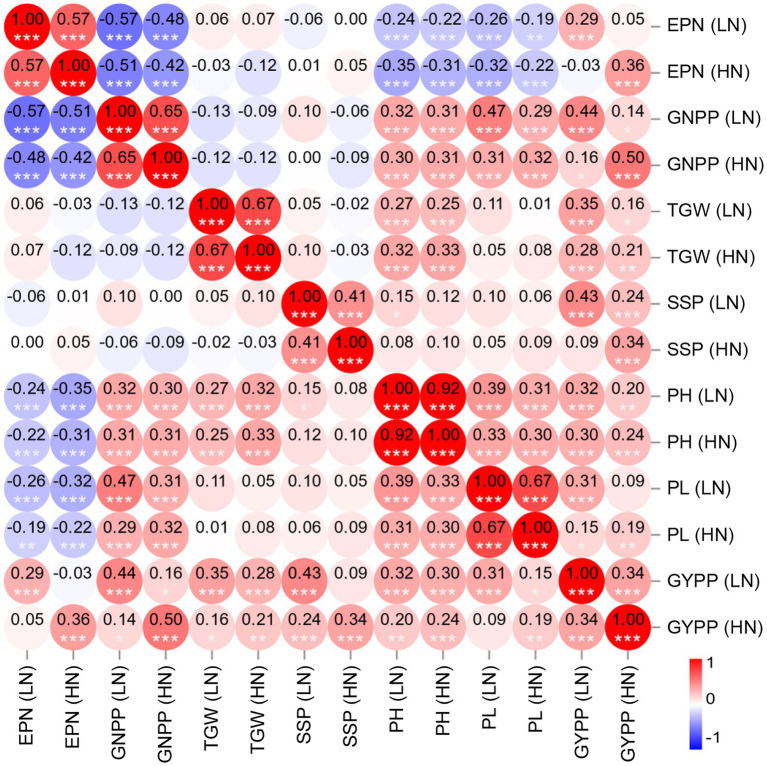
Heatmap of the correlations among the agronomic traits of the RIL population under two N levels. The color bar indicates the Pearson’s correlation coefficients, which are shown in the circles. ^***^p < 0.001, ^**^p < 0.01, and ^*^p < 0.05. EPN, effective panicle number; GNPP, grain number per panicle; TGW, thousand-grain weight; SSP, seed-setting percentage; PH, plant height; PL, panicle length; LN, low nitrogen; HN, high nitrogen.

To further examine the diverse effects of N levels on different agronomic traits as well as the interactions among these traits, we performed correlation analyses of the phenotypes. As expected, all the agronomic traits under HN conditions showed significant positive correlations with the corresponding traits under LN conditions, with the highest correlation obtained for PH (*R* = 0.92) and the lowest correlation obtained for GYPP (*R* = 0.34) followed by SSP (*R* = 0.41; Figure 2), which reflects the differing heritability of these traits. Significant positive correlations were also observed between GNPP and PH, GNPP and PL, TGW and PH, and PH and PL under either of the conditions (Figure 2). The EPN showed a significant negative correlation with each of GNPP, PH, and PL (Figure 2); the correlation coefficients between EPN and GNPP were as low as −0.57 (LN) and −0.42 (HN). The GYPP displayed a significant positive correlation with all examined agronomic traits under the same condition (Figure 2). We further examined the relationship between GYPP and four yield-related components using path analysis, and found that both EPN and GNPP mostly contributed to GYPP under both of the conditions as revealed by the path coefficients [0.80 and 0.77 for EPN under LN and HN conditions respectively; 0.91 and 0.91 for GNPP under LN and HN conditions, respectively (Supplementary Table S1)]. This result is consistent with the observation that large panicle numbers and large panicle sizes are two major factors related to high GYPP.

### Genetic map construction for the RIL population

After genotyping with an updated version of GeneChip Rice 44 K SNP microarrays, a total of 2,345 bin markers resulting from 12,152 effective SNPs were obtained to construct the linkage map for the RIL population, with an average of 195 markers per linkage group (chromosome; Supplementary Figures S1, S2). Chromosome 1 possessed the most markers (341), while chromosome 7 had the least (103). The total genetic distance of the linkage map was 1944.9 cM with an average interval of 0.8 cM between adjacent bins (Supplementary Figures S1, S2). The marker density (0.6 cM/marker) was highest on chromosome 12, while the lowest marker density (1.5 cM/marker) was detected on chromosome 7. Regarding the physical distances, the marker density was the highest on chromosome 2 (114.0 kb/marker) and the lowest (288.3 kb/marker) on chromosome 7, with an average interval of 158.7 kb (Supplementary Figures S1, S2). This high-resolution linkage map enables the precise mapping of QTLs.

### QTL mapping for agronomic traits under two N conditions

**Figure 3:**
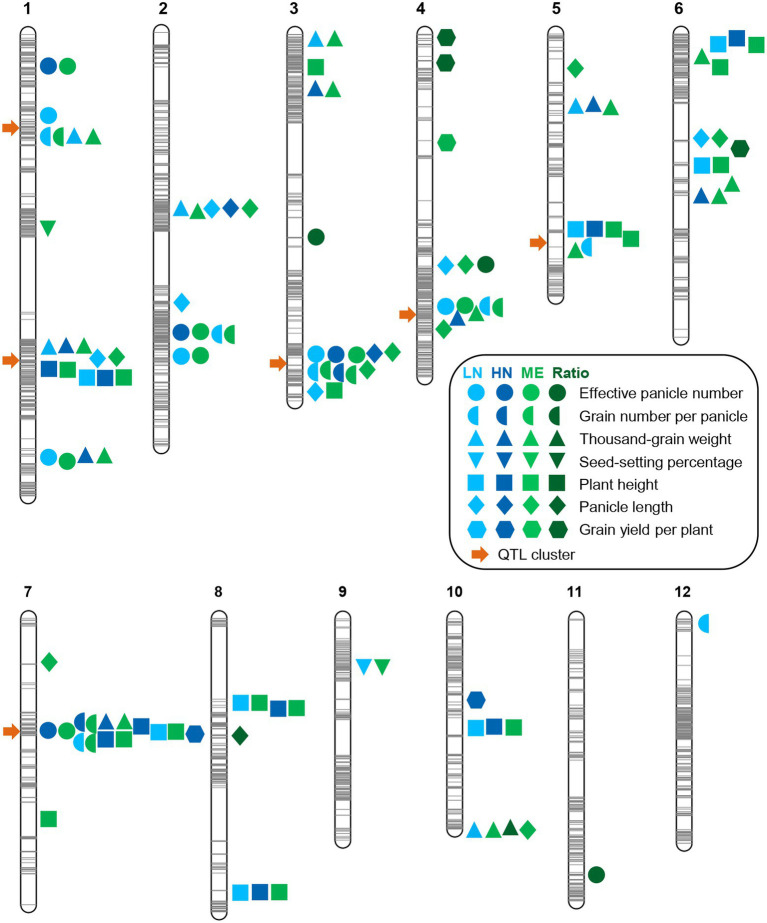
Summary of QTL locations on the linkage map of the RIL population. The gray lines on the chromosomes show the genetic positions of the markers. The light blue, dark blue, light green, and dark green symbols on the right of the chromosomes indicate the LN (low nitrogen), HN (high nitrogen), ME (multi-environment), and ratio trait-related QTLs, respectively. The orange arrows on the left of the chromosomes indicate QTL clusters.

Based on the above genetic map, we performed QTL analysis for these seven agronomic traits under both conditions using the ICIM approach (Meng et al., 2015). We identified a total of 33 QTLs (LOD ≥ 3.0) for these traits under LN conditions (Figure 3; Table 2). Among the five QTLs identified for EPN, two major ones (PVE ≥ 10%) with LOD values up to 16.70 and 10.75, respectively, explained 18.69% and 11.39% of PVE, and the additive effects were from the ZS97 alleles (Table 2). For the six QTLs of TGW, the additive effects of five were from the ZS97 alleles, and one major QTL with a LOD value of 12.03 explained 13.63% of PVE (Table 2). The additive effects of six of the seven QTLs for GNPP were from the HR5 alleles, and two major QTLs with LOD values up to 12.12 and 11.90 explained 11.36% and 11.19% of PVE, respectively (Table 2). The HR5 alleles also had additive effects for six of the eight QTLs for PH and five of the six QTLs for PL (Table 2). In contrast to the major QTL of PH (LOD, 15.04; PVE, 12.43%), the major QTL of PL (LOD, 19.68; PVE, 21.68%) showed a ZS97-derived additive effect (Table 2). Only one QTL was identified for SSP (Table 2).

**Table 2:** QTLs associated with seven agronomic traits of the RIL population under LN conditions.

ID	Trait^a^	Chr.^b^	Peak (cM)	L-Marker (position)^c^	R-Marker (position)^d^	Interval^e^	LOD^f^	PVE(%)^g^	Add^h^	Note^i^
*qEPN-LN1*	EPN	1	40.5	1_71 (4.96)	1_72 (5.22)	257.3	3.03	2.99	0.23	specific
*qEPN-LN2*	EPN	1	225.0	1_306 (41.01)	1_307 (41.7)	684.5	4.71	4.71	−0.29	specific
*qEPN-LN3*	EPN	2	170.8	2_265 (29.86)	2_266 (30.33)	474.0	4.44	4.55	0.29	specific
*qEPN-LN4*	EPN	3	169.6	3_239 (28.44)	3_240 (28.57)	124.8	16.70	18.69	0.59	common
*qEPN-LN5*	EPN	4	145.0	4_151 (31.07)	4_152 (31.14)	64.1	10.75	11.39	0.46	specific
*qGNPP-LN1*	GNPP	1	54.0	1_95 (6.63)	1_96 (6.67)	42.1	5.64	4.91	−9.89	specific
*qGNPP-LN2*	GNPP	2	159.7	2_236 (27.37)	2_237 (27.41)	47.1	6.45	5.66	−10.63	specific
*qGNPP-LN3*	GNPP	3	178.4	3_247 (29.83)	3_248 (29.91)	71.7	12.12	11.36	−15.05	common
*qGNPP-LN4*	GNPP	4	145.3	4_153 (31.2)	4_154 (31.24)	41.3	8.67	7.75	−12.62	specific
*qGNPP-LN5*	GNPP	5	111.0	5_85 (24.15)	5_86 (25.62)	1465.9	4.38	4.18	9.14	specific
*qGNPP-LN6*	GNPP	7	62.5	7_50 (9.18)	7_51 (16.11)	6934.1	11.90	11.19	−15.56	common
*qGNPP-LN7*	GNPP	12	2.1	12_6 (0.36)	12_7 (0.48)	120.9	3.08	2.62	−7.23	specific
*qTGW-LN1*	TGW	1	52.7	1_93 (6.5)	1_94 (6.63)	122.2	6.68	7.28	0.69	specific
*qTGW-LN2*	TGW	1	164.6	1_202 (31.71)	1_203 (31.74)	30.4	3.35	3.50	−0.49	common
*qTGW-LN3*	TGW	2	91.2	2_101 (14.08)	2_102 (15.6)	1520.5	5.62	5.99	0.63	specific
*qTGW-LN4*	TGW	3	3.7	3_9 (0.45)	3_10 (0.49)	43.9	4.27	4.50	0.56	specific
*qTGW-LN5*	TGW	5	37.1	5_27 (4.79)	5_28 (4.95)	162.0	12.03	13.63	0.95	common
*qTGW-LN6*	TGW	10	112.8	10_137 (23.03)	10_138 (23.07)	42.4	5.93	6.36	0.65	specific
*qSSP-LN1*	SSP	9	25.8	9_38 (10.26)	9_39 (10.35)	85.9	4.03	7.14	2.16	specific
*qPH-LN1*	PH	1	181.0	1_250 (35.11)	1_251 (35.13)	14.2	6.11	4.64	2.41	common
*qPH-LN2*	PH	5	104.8	5_81 (24.11)	5_82 (24.13)	20.6	4.68	3.53	−2.11	common
*qPH-LN3*	PH	6	7.2	6_26 (0.91)	6_27 (1.01)	94.7	6.20	4.73	2.44	common
*qPH-LN4*	PH	6	69.2	6_106 (11.48)	6_107 (11.64)	151.9	3.89	2.91	−1.92	specific
*qPH-LN5*	PH	7	60.4	7_39 (7.07)	7_40 (7.15)	78.0	15.04	12.43	−4.08	common
*qPH-LN6*	PH	8	45.1	8_15 (3.7)	8_16 (4.05)	345.9	3.92	3.02	−1.99	specific
*qPH-LN7*	PH	8	144.6	8_120 (27.34)	8_121 (27.6)	253.6	3.58	2.67	−1.85	common
*qPH-LN8*	PH	10	62.8	10_86 (16.9)	10_87 (17.13)	230.0	4.54	3.41	−2.08	common
*qPL-LN1*	PL	1	170.6	1_217 (32.94)	1_218 (32.99)	55.8	3.36	3.18	−0.50	specific
*qPL-LN2*	PL	2	92.3	2_107 (16)	2_108 (16.06)	59.6	19.68	21.68	1.28	common
*qPL-LN3*	PL	2	143.0	2_181 (23.56)	2_183 (23.7)	141.8	3.05	2.92	−0.47	specific
*qPL-LN4*	PL	3	190.8	3_272 (31.72)	3_273 (31.75)	29.4	3.70	3.52	−0.52	specific
*qPL-LN5*	PL	4	123.5	4_83 (25.75)	4_84 (26.18)	431.3	6.33	6.18	−0.68	specific
*qPL-LN6*	PL	6	57.2	6_104 (8.89)	6_105 (11.29)	2408.0	3.64	3.48	−0.51	specific

a EPN, effective panicle number; GNPP, grain number per panicle; TGW, thousand-grain weight; SSP, seed-setting percentage; PH, plant height; PL, panicle length.

b Chr, chromosome.

c L-Marker, ID of the left marker. The physical position (Mb) of the marker is given in the brackets.

d R-Marker, ID of the right marker. The physical position (Mb) of the marker is given in the brackets.

e Interval, the physical interval between the left and right marker.

f LOD, logarithm of odds.

g PVE (%), phenotypic variance explained (%).

h ADD, additive effect; positive and negative values, respectively, indicate positive alleles from ZS97 and HR5.

i Specific, LN-specific QTLs; common, common QTLs also detected under HN condition (co-localization).

We also identified 26 QTLs for all the agronomic traits under HN conditions, except for SSP, including four, two, seven, nine, two, and two QTLs for EPN, GNPP, TGW, PH, PL, and GYPP, respectively (Figure 3; Table 3). All of the EPN-related QTLs and four of the TGW-related QTLs, including one major QTL for each trait (LOD, 11.68 and 11.36; PVE, 16.72 and 10.67), showed additive effects from the ZS97 alleles (Table 3). All the GNPP-related QTLs, including a major one (LOD, 6.91; PVE, 11.64), possessed additive effects from the HR5 alleles. Seven of the PH-related QTLs and one of the PL-related QTLs also showed HR5-derived additive effects, but the additive effects of one major QTL for each of PH and PL (LOD, 38.73 and 10.92; PVE, 19.26 and 15.29) were derived from the ZS97 alleles (Table 3). The additive effects of two GYPP-related QTLs were from the HR5 alleles (Table 3).

**Table 3:** QTLs associated with seven agronomic traits of the RIL population under HN conditions.

ID	Trait^a^	Chr.^b^	Peak (cM)	L-Marker (position)^c^	R-Marker (position)^d^	Interval^e^	LOD^f^	PVE(%)^g^	Add^h^	Note^i^
*qEPN-HN1*	EPN	1	18	1_36 (2.36)	1_37 (2.37)	13.5	3.8	5.03	0.3	specific
*qEPN-HN2*	EPN	2	158.4	2_232 (27.11)	2_233 (27.19)	72.2	4.45	5.94	0.33	specific
*qEPN-HN3*	EPN	3	169.6	3_239 (28.44)	3_240 (28.57)	124.8	11.68	16.72	0.55	common
*qEPN-HN4*	EPN	7	56.3	7_26 (5.73)	7_29 (5.97)	244.4	4.16	5.54	0.32	specific
*qGNPP-HN1*	GNPP	3	182.8	3_260 (30.54)	3_262 (30.56)	15.5	6.91	11.64	−13.33	common
*qGNPP-HN2*	GNPP	7	56	7_25 (5.65)	7_27 (5.83)	175.6	3.56	5.81	−9.59	common
*qTGW-HN1*	TGW	1	163.7	1_201 (31.68)	1_202 (31.71)	29	5.4	4.95	−0.6	common
*qTGW-HN2*	TGW	1	221.2	1_306 (41.01)	1_307 (41.7)	684.5	8.78	8.09	0.74	specific
*qTGW-HN3*	TGW	3	28.1	3_81 (3.43)	3_82 (3.45)	15.7	5.86	5.25	0.6	specific
*qTGW-HN4*	TGW	4	150.9	4_173 (32.37)	4_174 (32.6)	228.6	5.18	4.67	0.57	specific
*qTGW-HN5*	TGW	5	36.7	5_25 (4.73)	5_27 (4.79)	56.4	11.36	10.67	0.85	common
*qTGW-HN6*	TGW	6	84.4	6_123 (19.5)	6_125 (19.74)	245.6	3.94	3.45	−0.48	specific
*qTGW-HN7*	TGW	7	56.5	7_29 (5.97)	7_30 (6.01)	40.5	7.31	6.6	−0.68	specific
*qPH-HN1*	PH	1	178.5	1_240 (34.79)	1_241 (34.79)	2.6	22.27	9.43	−4.85	specific
*qPH-HN2*	PH	1	181.1	1_251 (35.13)	1_252 (35.16)	26.8	38.73	19.26	6.92	common
*qPH-HN3*	PH	5	105.2	5_82 (24.13)	5_83 (24.15)	19.2	3.93	1.4	−1.87	common
*qPH-HN4*	PH	6	4.6	6_18 (0.66)	6_19 (0.69)	26.5	6.15	2.24	2.36	common
*qPH-HN5*	PH	7	56.9	7_31 (6.05)	7_32 (6.36)	314.1	8.32	3.1	−2.85	specific
*qPH-HN6*	PH	7	62.3	7_50 (9.18)	7_51 (16.11)	6934.1	9.07	3.41	−3.04	common
*qPH-HN7*	PH	8	50.1	8_19 (4.43)	8_20 (4.54)	112.7	10.18	3.85	−3.17	specific
*qPH-HN8*	PH	8	144.5	8_119 (27.28)	8_120 (27.34)	64.7	4.64	1.67	−2.06	common
*qPH-HN9*	PH	10	62.1	10_85 (14.91)	10_86 (16.9)	1987.6	7.75	2.95	−2.72	common
*qPL-HN1*	PL	2	92.1	2_104 (15.66)	2_105 (15.84)	177.2	10.92	15.29	0.91	common
*qPL-HN2*	PL	3	165.3	3_220 (27.29)	3_221 (27.39)	94.4	3.19	4.15	−0.48	specific
*qGYPP-HN1*	GYPP	7	61.1	7_45 (7.94)	7_46 (8.03)	90.7	4.05	6.96	−1.41	specific
*qGYPP-HN2*	GYPP	10	46.5	10_75 (14.09)	10_76 (14.45)	360.4	3.46	6.02	−1.27	specific

a EPN, effective panicle number; GNPP, grain number per panicle; TGW, thousand-grain weight; SSP, seed-setting percentage; PH, plant height; PL, panicle length; GYPP, grain yield per plant.

b Chr, chromosome.

c L-Marker, ID of the left marker. The physical position (Mb) of the marker is given in the brackets.

d R-Marker, ID of the right marker. The physical position (Mb) of the marker is given in the brackets.

e Interval, the physical interval between the left and right marker.

f LOD, logarithm of odds.

g PVE (%), phenotypic variance explained (%).

h ADD, additive effect; positive and negative values, respectively, indicate positive alleles from ZS97 and HR5.

i Specific, HN-specific QTLs; common, QTLs also detected under LN condition (co-localization).

Comparison of the QTLs detected under the two conditions indicated that 12 QTLs under HN conditions were co-localized with their counterparts under LN conditions, including one QTL for EPN, two for GNPP, two for TGW, six for PH, and one for PL (Figure 3; Tables 2, 3). Nearly all major QTLs showed co-localization between the conditions, except for a major EPN-related QTL under LN conditions (Tables 2, 3). This comparison also identified 21 LN-specific QTLs (four for EPN, five for GNPP, one for SSP, four for TGW, two for PH, and five for PL) and 14 HN-specific QTLs (three for EPN, five for TGW, three for PH, one for PL, and two for GYPP; Tables 2, 3). These condition-specific QTLs may be partly involved in the responses to different N levels.

### Multi-environment QTLs and QTL clusters

To further examine the genetic basis of NUE, we performed QTL-by-environment interaction (QEI) analysis (LN and HN environments). We identified 57 multi-environment QTLs, including 7 for EPN, 7 for GNPP, 14 for TGW, 2 for SSP, 16 for PH, 10 for PL, and 1 for GYPP (Figure 3; Supplementary Table S2). In addition to our finding that most of the aforementioned single-environment QTLs (except five QTLs) were detected in the QEI analysis, two for TGW, one for SSP, four for PH, four for PL, and one for GYPP were newly identified (Figure 3; Supplementary Table S2). There were in total eight QTLs with LOD scores for additive by environment effects >3.0 (Supplementary Table S2), comprising three for GNPP, two for TGW, and three for PH, which might be related to the N response.

We also performed QTL mapping using ratio traits (relative ratios of LN to HN for each of the agronomic traits). All the ratio traits of the RIL population displayed normal distributions (Supplementary Figure S3; Supplementary Table S3). Eight QTLs were identified, comprising three for EPN, one for TGW, one for PL, and three for GYPP (Figure 3; Supplementary Table S4). One EPN-related QTL and two GYPP-related QTLs showed additive effects from the HR5 alleles, while the other QTLs possessed those from the ZS97 alleles (Supplementary Table S4).

Combining these comprehensive QTL analysis results, we observed six QTL clusters (≥ 3 traits), including two on chromosome 1 and one on chromosomes 3, 4, 5, and 7 (Figure 3). These QTL clusters were mainly related to EPN, GNPP, TGW, and PH (Figure 3; Supplementary Figure S4). The largest cluster in chromosome 7 comprised 13 QTLs related to five traits (Figure 3). Among each pair between the seven agronomic traits, the pairs between EPN and GNPP, and between GNPP and TGW, showed the highest frequency in these QTL clusters (each in four clusters; Figure 3; Supplementary Figure S4). These pairs of agronomic traits generally showed a negative correlation—the so-called trade-offs between traits.

For all QTLs identified, the QTL regions (the physical distance between the flanking markers of a QTL) were as small as 512 kb on average; more than half (50.81%) of the QTLs spanned an interval smaller than 100 kb; and one third (29.03%) spanned intervals smaller than 50 kb (Supplementary Figure S5). The narrow regions of these QTLs indicate the possibility that the candidate genes can be easily cloned.

### Candidate genes of an EPN-related QTL for both LN and HN conditions

**Figure 4:**
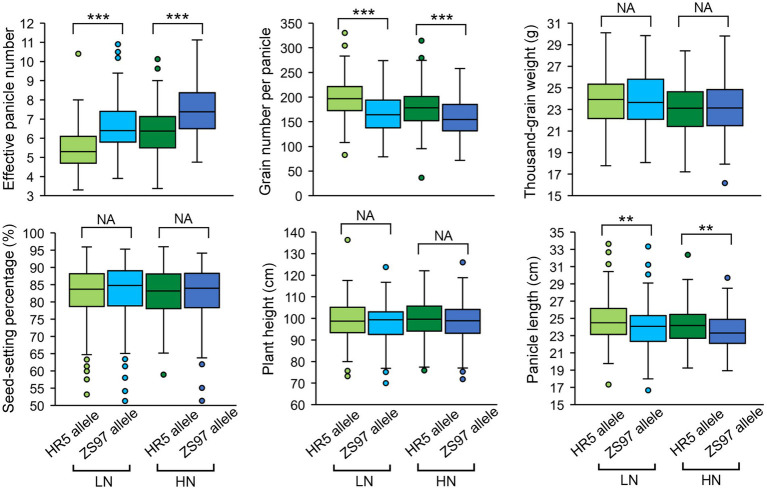
Performance of the *qEPN-LN4*/*qEPN-HN3* alleles in the RIL population. ^***^p < 0.001, ^**^p < 0.01; NS, not significant. LN, low nitrogen; HN, high nitrogen.

**Figure 5:**
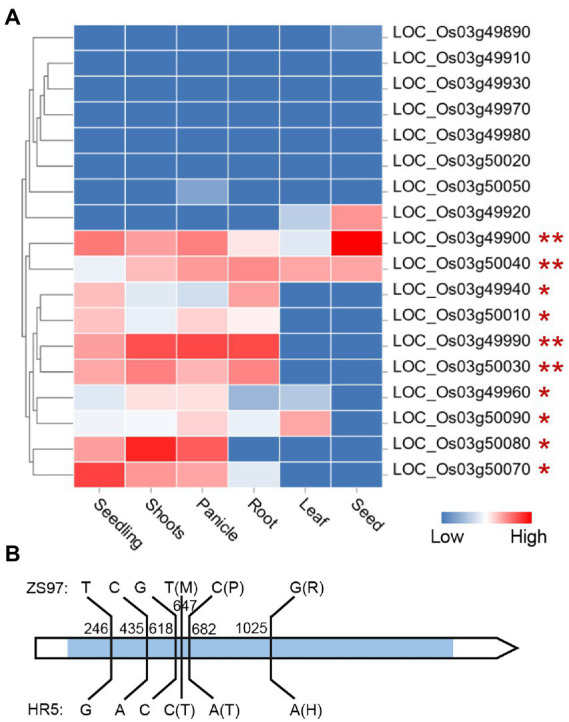
Expression profile of the genes in the *qEPN-LN4*/*qEPN-HN3* locus in different tissues **(A)** and sequence variations between the *SLR1* alleles of the parents **(B)**. The color bar indicates the relative expression levels. The genes are clustered according to their expression profiles. The genes expressed in both or either the panicle and root are marked by ** or *. The blue rectangle in the gene model indicates the coding region. The relative positions to the start codon of the variations are shown. The amino acid changes induced by non-synonymous mutations are indicated in the brackets.

There was one EPN-related QTL (*qEPN-LN4*/*qEPN-HN3*) detected under both LN and HN conditions, which was located in the QTL cluster on chromosome 3 (Figure 3; Tables 2, 3). We grouped the RILs into two groups according to the alleles of *qEPN-LN4*/*qEPN-HN3* and found significant differences in EPN, GNPP, and PL under either of the conditions between the two groups (Figure 4). The ZS97 allele positively impacted EPN, but negatively impacted GNPP and PL (Figure 4). According to the physical positions of the flanking markers, the QTL region spanned 124.80 kb from 28.44 Mb to 28.57 Mb and contained 18 annotated genes (Supplementary Table S5). We examined the tissue expression profiles of these genes and found four genes showing relatively high expression levels in both the roots and panicles, and six in either of the roots or panicles (Figure 5A). One of them (LOC_Os03g49990), which was highly expressed in both the roots and panicles and that encodes a DELLA protein SLR1, was recently reported to regulate TN and NUE (Li et al., 2018; Liao et al., 2019; Wu et al., 2020). Sequencing of the *SLR1* alleles of the parents identified six SNPs in the coding region, among which three resulted in amino acid changes (Figure 5B; Supplementary Datasheet S1).

### Candidate genes of an EPN-related QTL for HN conditions only

**Figure 6:**
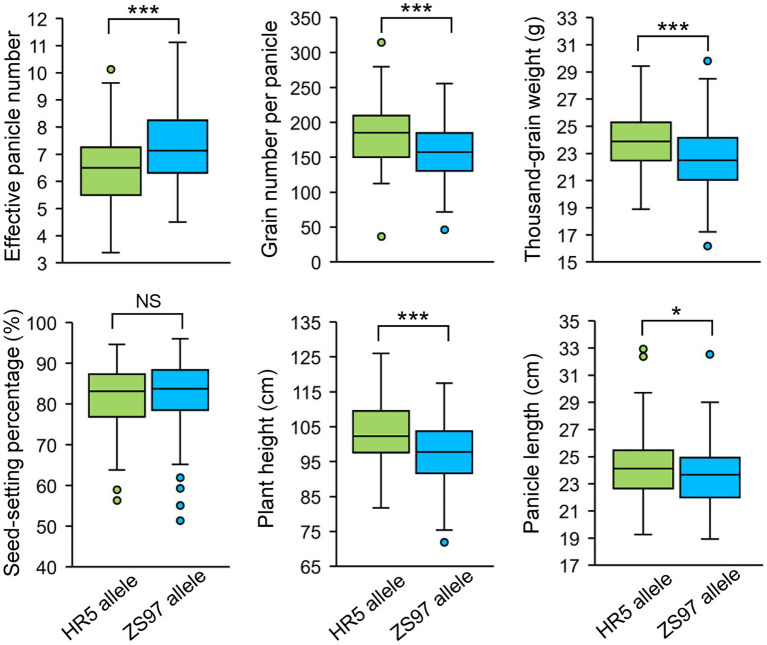
Performance of the *qEPN-HN4* alleles in the RIL population under HN conditions. ^***^p < 0.001, ^*^p < 0.05; NS, not significant.

**Figure 7:**
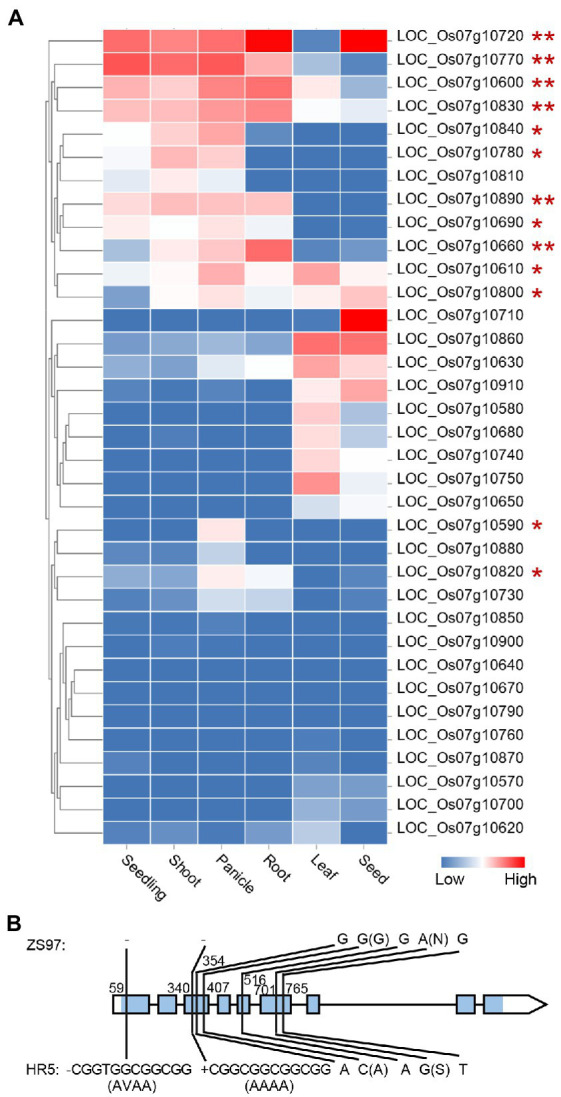
Expression profile of the genes in the *qEPN-HN4* locus in different tissues **(A)** and sequence variations between the *OsbZIP59* alleles of the parents **(B)**. The color bar indicates the relative expression levels. The genes are clustered according to their tissue expression profiles. The genes expressed in both or either the panicle and root are marked by ** or *. The blue rectangle in the gene model indicates the coding region. The relative positions to the start codon of the variations are shown. The amino acid changes induced by non-synonymous mutations are indicated in the brackets. Only the variations in the exons are displayed.

The HN-specific EPN-related QTL *qEPN-HN4* was located in the QTL cluster in chromosome 7 (Figure 3; Tables 2, 3). The two groups of RILs with the HR5 or ZS97 alleles of *qEPN-HN4* showed significant differences in five traits under HN conditions (Figure 6). The ZS97 allele had a positive effect on EPN but a negative effect on GNPP, TGW, PH, and PL (Figure 6). The physical positions of the flanking markers of *qEPN-HN4* spanned a region of 244 kb from 5.72 to 5.97 Mb, containing 35 annotated genes (Supplementary Table S6). The tissue expression profiles of these genes showed that six genes were relatively highly expressed in both the roots and panicles and seven were highly expressed in the roots (Figure 7A).

**Figure 8:**
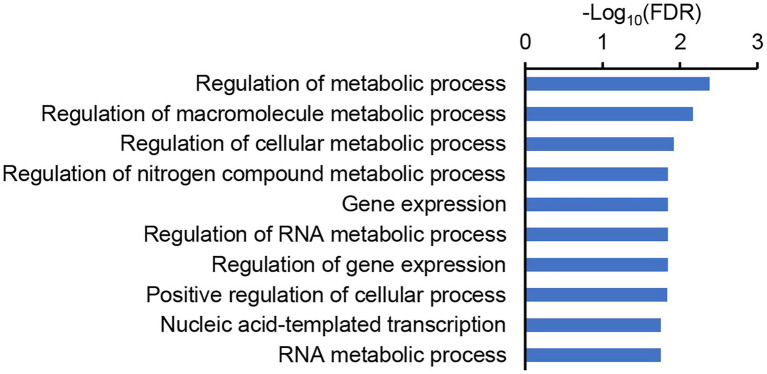
The GO enrichment of the co-expressed genes of *OsbZIP59*. The top-10 GO enriched items of biological process are shown, and the false discovery rate (FDR) is log_10_-transferred. The full results of the GO enrichment are provided in Supplementary Table S8.

We then examined the annotation and homology of these genes, and found that *LOC_Os07g10890*, one of the genes that was highly expressed in both the roots and panicles, encodes the transcription factor OsbZIP59, which is homologous to bZIP16 in *A. thaliana* targeting multiple NUE-related genes, including *NRT3.1* (Gaudinier et al., 2018). Sequencing of the *OsbZIP59* alleles of the parents revealed five SNPs and two 12-bp indels in the coding region (Figure 7B; Supplementary Datasheet S1). Two of the SNPs were non-synonymous mutations, and each of the indels induced insertion/deletions of four amino acids (Figure 7B). To further test the possibility that *OsbZIP59* is the candidate gene of *qEPN-HN4*, we performed co-expression network analysis considering its function as a transcription factor of the bZIP family. We used the top-50 co-expressed genes to construct the co-expression network (Supplementary Table S7) and subjected these genes to GO enrichment analysis. The GO terms related to metabolic processes including a term of “regulation of nitrogen compound metabolic process” (ranked fourth) were significant enriched (FDR < 0.05), and those related to gene expression were also enriched (Figure 8). Therefore, we suggest that *OsbZIP59* is a possible candidate gene of *qEPN-HN4*.

### A valuable resource for the QTL mapping for NUE

To promote the use of our QTL mapping results, the genes in the regions of all QTLs with <500 kb intervals are provided in Supplementary Datasheet S2. We further analyzed the possible candidate genes of the common QTLs detected under both LN and HN conditions, as well as those of the ratio trait-related QTLs. Through examinations of the tissue expression profiles and functional annotations, we successfully predicted the possible candidate genes for three of the examined QTLs. Two genes in the *qTGW-LN2*/*qTGW-HN1* locus for TGW showed relatively high expression levels in the grain, and one of them (*LOC_Os01g55110*) encodes the RING-type E3 ubiquitin ligase NBIP1 known to interact with NRT1.1B (Supplementary Table S9; Hu et al., 2019). In the *qTGW-RT1* locus for the ratio trait of TGW, one gene (*LOC_Os10g41430*) encodes a cyclin protein CYC U4; 1 that is highly expressed in the grain (Supplementary Table S9). In the *qPH-LN7*/*qPH-HN8* locus for PH, one of the genes expressed in the shoots (*LOC_Os08g43130*) encodes the NCK-associated protein 1 (NAP1)-like protein LPL3 involved in cell morphogenesis (Supplementary Table S9), and its mutant showed reduced PH (Zhou et al., 2016).

## Discussion

The NUE trait is a complex quantitative trait involved in multiple biological processes and agronomic traits; however, the NUE regulatory network remains largely unknown. We used a high-resolution genetic map to perform comprehensive QTL analyses of seven key agronomic traits under two different N levels. Considering the issue of QTL co-localization, we identified 11 non-redundant QTLs for EPN, 7 for GNPP, 13 for TGW, 2 for SSP, 15 for PH, 12 for PL, and 6 for GYPP (Tables 2, 3; Supplementary Tables S2, S4). Among them, seven major non-redundant QTLs were identified for the examined traits (Tables 2, 3; Supplementary Table S2). Several major QTLs were co-localized with known cloned QTLs, such as *qTGW-LN5*/*qTGW-HN5*, which was co-localized with *qSW5*/*GW5*/*GSE5,* which is related to TGW and grain width (Shomura et al., 2008; Duan et al., 2017; Liu et al., 2017). These findings confirmed the effectiveness of our QTL mapping. In the QEI analysis, most of the single-environment QTLs were detected, and 12 new QTLs were newly identified (Supplementary Table S2), suggesting that the QEI analysis was an important complementary analysis in our study.

The RIL population derived from ZS97 and HR5 was previously genotyped with simple sequence repeat (SSR) markers. Tong et al. (2011) used 245 SSR markers from 188 lines of the RIL population to map the QTLs for grain yield and its components [GYPP, panicle number per plant (PNPP), GNPP, filled grains per panicle (FGPP), spikelet fertility percentage (SFP), and 100-grain weight (HGW)] under three N levels (0, 150, and 300 kg urea/ha). In this study, 2,345 bin markers were obtained from SNP microarrays for 261 lines. The average interval between two bin markers was only 158.7 kb, which is similar to the previously reported linkage disequilibrium decay of *indica* subspecies (Huang et al., 2010; Zhao et al., 2011), enabling the precise identification of QTL locations and candidate genes. Among the six agronomic traits we examined under two N levels (120 and 300 kg urea/ha), PH and PL were not examined in Tong et al. (2011), and the other traits were similar (PNPP vs. EPN, HGW vs. TGW) or the same (GNPP, SFP/SSP, GYPP) as in this previous study. However, among the 57 QTLs (41 non-redundant QTLs) that Tong et al. (2011) discovered based on a mixed linear model, only three PNPP-related QTLs, two HGW-related QTLs, and one GYPP-related QTL were co-localized with the QTLs (EPN-, TGW-, and GYPP-related, respectively) in our study based on ICIM. A reason for this difference could be that Tong et al. (2011) used the averages of the traits obtained from different regions of distinct photoperiod and climatic conditions. Remarkably, no QTL was detected for SFP by Tong et al. (2011), while we identified two non-redundant QTLs for SSP. In particular, the QTL regions we determined were small (512 kb on average), and more than half of the QTLs spanned an interval smaller than 100 kb (Tables 2, 3; Supplementary Tables S2, S3; Supplementary Figure S5). Consequently, our results provide the precise mapping of NUE-related QTLs, promoting the cloning of candidate genes (discussed in detail below).

Tong et al. (2011) found that SFP had the greatest contribution to yield at N levels of 150 and 300 kg urea/ha, whereas FGPP contributed the most to yield at an N level of zero. However, we observed no response of SSP to different N levels (120 and 300 kg urea/ha). Our path analysis revealed major contributions of both EPN and GNPP to GYPP (Supplementary Table S1). Recent research found that a high NUE for yield is associated with a positive role of N in promoting TN, which consequently increases the total grain number per plant and thus improves yield with less N fertilizer (Sun et al., 2014; Wu et al., 2020; Liu et al., 2021). Consistent with this, we also observed that EPN was a major response of the population to N levels. Interestingly, the RIL population showed extreme phenotypical segregation of EPN, although both the parents exhibited a similar performance of EPN (Figure 1), suggesting distinct genetic loci controlling the EPN of the parents. In line with this possibility, we identified a total of 11 non-redundant EPN-related QTLs. Although several TN-related QTLs responding to N levels have been cloned (Sun et al., 2014; Liu et al., 2021), most of these EPN-related QTLs have yet to be cloned (only *qEPN-LN3* is co-localized with *qNGR2*; Li et al., 2018), suggesting that much about rice NUE regulation remains to be investigated.

Although the population we used is an RIL population, the narrow regions of most QTLs allow for candidate gene analyses. To validate the value of the resource we presented, we demonstrated five cases of candidate gene analysis. The possible candidate gene of *qEPN-LN4*/*qEPN-HN3* was predicted to encode a DELLA protein, SLR1, which has been recently reported to regulate TN through interacting with a TN regulator MOC1 to inhibit the degradation of the latter (Liao et al., 2019). SLR1 also interacts with and inhibits the transcription factor GRF4, which works with the transcription coactivator GIF1 and another transcription factor MYB61 to regulate multiple N metabolism genes (Li et al., 2018; Gao et al., 2020). It is also reported that SLR1 interacts with an NUE and TN regulator, namely NGR5, to protect its degradation by the gibberellin receptor GID1 (Wu et al., 2020). Although the known function of SLR1 and its multiple reported mutants has been discussed (Ikeda et al., 2001; Asano et al., 2009; Hayashi-Tsugane et al., 2011; De Vleesschauwer et al., 2016; Jin et al., 2022), the natural variations or elite alleles have not been identified to date. We identified three missense SNPs of *SLR1* between the two parents, which might be the causal mutations of the alleles. The possible candidate gene of *qEPN-HN4* is inferred to encode the transcription factor OsbZIP59. We found multiple lines of evidence suggesting the possibility that OsbZIP59 is involved in NUE. Additionally, OsbZIP59 has been previously screened as a brassinosteroid receptor kinase BRI1-interacting protein (Hirabayashi et al., 2004). Several NUE-related genes are known to be associated with BR signaling (Sakamoto et al., 2006; Che et al., 2015; Li et al., 2018; Wu et al., 2020; Liu et al., 2021). The possible candidate genes of *qTGW-LN2*/*qTGW-HN1* and *qTGW-RT1* were predicted to encode the RING-type E3 ubiquitin ligase NBIP1 and cyclin protein CYC U4; 1, respectively. NBIP1 is known to be highly expressed in the roots and leaf blades and is responsible for degradation of the phosphate signaling repressor SPX4 by interacting with the nitrate sensor NRT1.1B, which integrates nitrogen and phosphorus signaling (Hu et al., 2019). We found that NBIP1 was also highly expressed in the grain and could regulate TGW. CYC U4; 1 controls cell proliferation and is regulated by BR signaling at both the gene expression and protein levels through the transcription factor BES1/BZR1 and kinase GSK3, respectively. BR signaling modulates grain sizes as well as NUE, and its multiple components have been reported to regulate TGW, including BES1/BZR1, GSK3, and another cyclin protein, CycT1; 3 (Qi et al., 2012; Li et al., 2019; Gao et al., 2019a). The candidate gene of *qPH-LN7*/*qPH-HN8*, *LPL3*, encodes a component of the SCAR/WAVE complex involved in actin nucleation and function, which controls epidermal cell morphogenesis by organizing F-actin; a T-DNA insertion mutant of *LPL3* showed moderate dwarfism (Zhou et al., 2016). However, the relationship between microfilaments and NUE is unclear. The candidate genes of these QTLs still require further investigation using transgenes of different alleles in the future.

We identified six large QTL clusters mainly related to EPN, GNPP, TGW, and PH (Figure 3; Supplementary Figure S4), which might be related to the trade-offs between traits. Such QTL clusters could be a result of the tight linkage of several QTLs regulating different traits, or alternatively, the pleiotropic effect of a single QTL. Interestingly, the two major EPN-related QTLs were located in two QTL clusters. A recent principal component (PC) analysis on eight typical traits related to the plant architecture of *japonica* rice varieties revealed that the first PCs indicated a trade-off between panicle sizes and numbers, and a GWAS with the PC scores identified the known genes *NAL1* and *OsGATA28,* as well as a new gene *OsSPY*, the protein of which activates SLR1 (Yano et al., 2019). The possible candidate gene of *qEPN-LN4*/*qEPN-HN3*, namely *SLR1*, is related to gibberellin signaling and exerts pleiotropic effects, and additionally, SLR1 also works as a transcription factor of the GRAS-domain family and regulates many physiological processes as well as development (Ikeda et al., 2001; Fukao and Bailey-Serres, 2008; Huang et al., 2015; De Vleesschauwer et al., 2016; Lu et al., 2020; Mo et al., 2020; Jin et al., 2022). The molecular function of OsbZIP59 as a transcription factor may also have a pleiotropic effect on different traits by targeting different downstream genes. Accordingly, the pleiotropy of the causal genes may partly contribute to the QTL clusters; however, the genetic linkage of different QTLs cannot be completely excluded.

In addition to the increase of TN and EPN, HN has distinct impacts on different agronomic traits of different varieties, and even opposite effects (Mae, 1997). The six trait-related QTLs with narrow candidate regions identified herein provide a convenient avenue for causal gene cloning, which may help in the elucidation of the complex network of NUE and promote higher yield with less N input. Additionally, the high-resolution genetic map for this population also provides a valuable resource for the QTL mapping of other important and complex traits in *indica* subspecies.

## Data availability statement

The raw data of the effective SNPs of the population and parents have been deposited in Figshare (https://doi.org/10.6084/m9.figshare.20411631.v1).

## Author contributions

ST and SC designed the research, conceived and supervised the project, and drafted and revised the manuscript. XL, HJ, JY, JH, and MJ performed the experiments. XL, HJ, MJ, SC, and ST analyzed the data. HZ and LC provided analysis tools and materials. All authors contributed to the article and approved the submitted version.

## Funding

This work was supported by the National Natural Science Foundation (U19A2025, 32000377, 32172037, and 31870229), the Strategic Priority Research Program of the Chinese Academy of Sciences (XDA24010404), the Fundamental Research Funds for the Central Universities (KJQN202103), and the Open Project of National Key Laboratory of Plant Molecular Genetics.

## Conflict of interest

The authors declare that the research was conducted in the absence of any commercial or financial relationships that could be construed as a potential conflict of interest.

## Publisher’s note

All claims expressed in this article are solely those of the authors and do not necessarily represent those of their affiliated organizations, or those of the publisher, the editors and the reviewers. Any product that may be evaluated in this article, or claim that may be made by its manufacturer, is not guaranteed or endorsed by the publisher.

## Supplementary material

SUPPLEMENTARY DATASHEET S1 Sequences of the SLR1 and OsbZIP59 alleles of the parents.

SUPPLEMENTARY DATASHEET S2 List of genes in all QTLs with <500 kb intervals.
