# Supplementary figures and images for: Comprehensive QTL analyses of nitrogen use efficiency in indica rice
Source: Front Plant Sci. 2022 Sep 23;13:992225. doi: 10.3389/fpls.2022.992225 (PMC9539535; doi:10.3389/fpls.2022.992225)

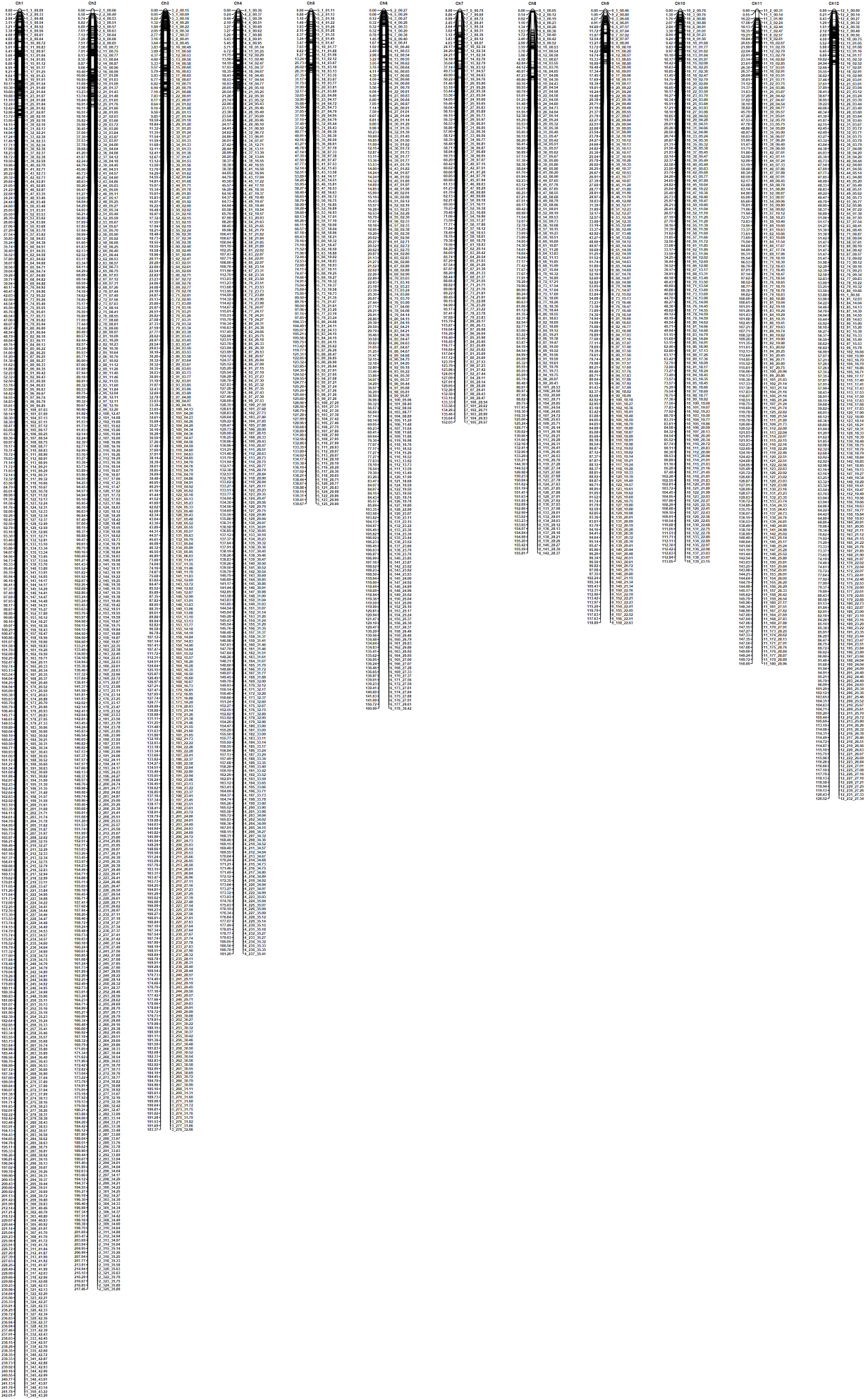

Supplementary Figure S1. The linkage map and bin markers of the RIL population.

Supplement: Supplementary file 3 [file Image_1.PDF]

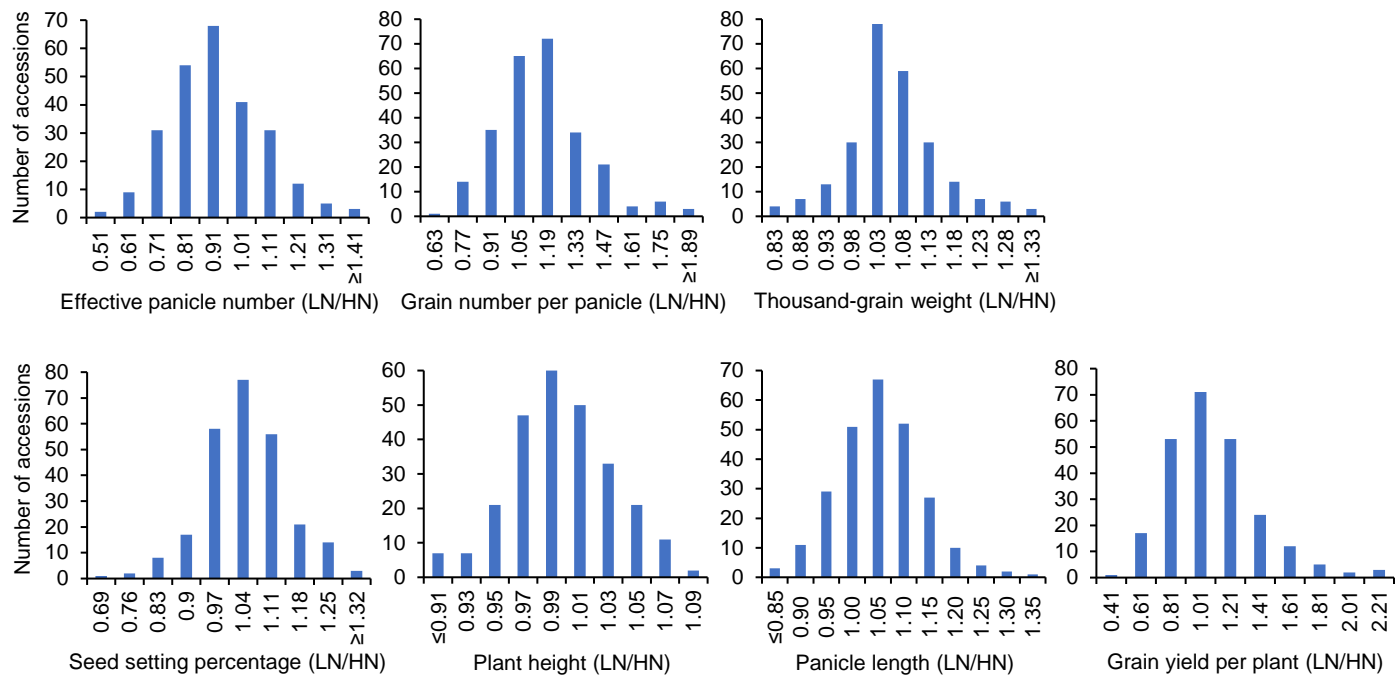

**Supplementary Figure S3. Performance of the ratio traits of the RIL population.**

Supplement: Supplementary file 5 [file Image_3.PDF]
