# Supplementary material for: Comprehensive QTL analyses of nitrogen use efficiency in indica rice
Source: Front Plant Sci. 2022 Sep 23;13:992225. doi: 10.3389/fpls.2022.992225 (PMC9539535; doi:10.3389/fpls.2022.992225)
Supplement: Supplementary file 4 [file Image_2.PDF]

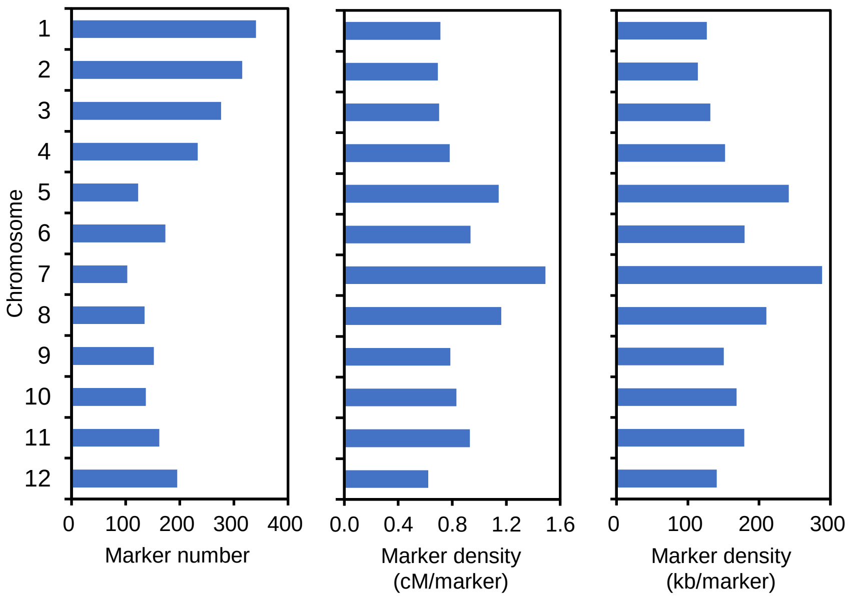

Supplementary Figure S2. Distribution of bin markers on the chromosomes. The number, genetic density, and physical density of the bin markers on different chromosomes are shown in the left, middle, and right panels, respectively.
