# Supplementary material for: Comprehensive QTL analyses of nitrogen use efficiency in indica rice
Source: Front Plant Sci. 2022 Sep 23;13:992225. doi: 10.3389/fpls.2022.992225 (PMC9539535; doi:10.3389/fpls.2022.992225)
Supplement: Supplementary file 6 [file Image_4.PDF]

	Trait	Cluster1	Cluster2	Cluster3	Cluster4	Cluster5	Cluster6	Frequency
●	EPN	YES	NO	YES	YES	NO	YES	4
◖	GNPP	YES	NO	YES	YES	YES	YES	5
▲	TGW	YES	YES	NO	YES	YES	YES	5
▼	SSP	YES	NO	NO	NO	NO	NO	1
■	PH	NO	YES	YES	NO	YES	YES	4
◆	PL	NO	YES	YES	YES	NO	NO	3
⬢	GYPP	NO	NO	NO	NO	NO	YES	1
	Trait NO.	4	3	4	4	3	5	

Supplementary Figure S4. The overlap of different agronomic traits in different related QTL clusters. EPN, effective panicle number; GNPP, grain number per panicle; TGW, thousand-grain weight; SSP, seed-setting percentage; PH, plant height; PL, panicle length; GYPP, grain yield per plant.
