# Supplementary material for: Comprehensive QTL analyses of nitrogen use efficiency in indica rice
Source: Front Plant Sci. 2022 Sep 23;13:992225. doi: 10.3389/fpls.2022.992225 (PMC9539535; doi:10.3389/fpls.2022.992225)
Supplement: Supplementary file 7 [file Image_5.PDF]

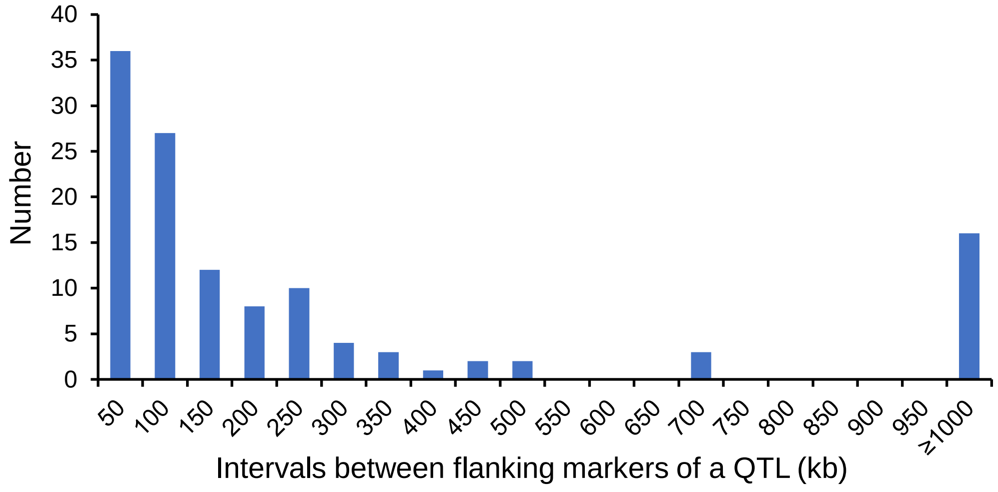

Supplementary Figure S5. Distribution of the intervals between the flanking markers of the QTLs.
